# Vb Cyclones Synchronized With the Arctic‐/North Atlantic Oscillation

**DOI:** 10.1029/2018JD029420

**Published:** 2019-03-26

**Authors:** M. Hofstätter, G. Blöschl

**Affiliations:** ^1^ Department of Climate Research Central Institute for Meteorology and Geodynamics Vienna Austria; ^2^ Vienna Doctoral Programme on Water Resource Systems Vienna University of Technology Austria; ^3^ Institute of Hydraulic Engineering and Water Resources Management Vienna University of Technology Austria

**Keywords:** mid latitude cyclones, clustering, Vb track, teleconnection, climate change, Western Mediterranean

## Abstract

Vb cyclones typically emerge in the Western Mediterranean and propagate to the Northeast into Central Europe. This paper explores the temporal characteristics of Vb cyclone occurrence based on cyclone tracks identified at the atmospheric levels of Z700 and sea level pressure, using JRA‐55 reanalysis data for the period 1959–2015. The risk of Vb occurrence was significantly high in the 1960s and has remained at a lower level since then. Vb cyclones do not occur fully randomly according to a Poisson point process. Eleven well‐separated and distinct clusters as well as 11 hiatus periods are identified, with average occurrence rates of 21.5 and 5.2 yrea^−1^, respectively. During the event of Vb, the large‐scale atmospheric circulation is changed into a state favoring the development of successive Vb cyclones. Clustering is very prominent in the case of Genoan Vb cyclones in summer as well as those Vb cyclones developing over the Iberian Peninsula or the North African Coast in winter. Superposition of the polar and the subtropical jet stream over the Western Mediterranean is identified as a main feature at the onset of Vb cyclones. Vb cyclone occurrence appears to be synchronized with the Northern Atlantic Oscillation (NAO; at Z500) and Arctic Oscillation (AO; at Z1000). Clusters have occurred when both NAO and AO were negative. This relation applies to Western Mediterranean cyclones not following a Vb track as well, however to a much weaker extent. In contrast, Vb cyclone frequency was particularly low from 1988 to 1997 during a sustained positive phase of both NAO and AO.

## Key Points

- In the past 50 years, the frequency of Vb cyclones was high in the 1960s and lower since then
- Vb cyclones were strongly synchronized with NAO and AO; high frequency clusters occurred when NAO and AO were negative
- The coupling of the polar and the subtropical jet stream over the Western Mediterranean is a driving mechanism of the onset of Vb cyclones

## 1 Introduction

Extreme weather events in Central Europe, such as large‐scale heavy precipitation and wind storms, are often related to the passage of midlatitude cyclones (Pfahl & Wernli, 2012; Hofstätter et al., 2018, H18 hereafter; Donat et al., 2011; Leckebusch et al., 2006). These types of events, respectively, account for about 53% and 15% of the economic losses due to natural hazards in Germany (Munich Re, 1999) together with injuries and loss of life. Atmospheric research has traditionally prioritized wind storms, whereas large‐scale heavy precipitation events have gained increasing attention only after the 2002 summer floods in Central Europe (Grazzini & van der Grijn, 2002; Ulbrich et al., 2003a, 2003b) that were caused by Vb cyclones (van Bebber, 1891). Historically, Vb cyclones have been vaguely described as low pressure systems propagating from the Western Mediterranean (WM) Sea to the Northeast, by crossing Northern Italy and leaving the Alpine ridge on the left. In recent years, it has become obvious that Vb cyclones are highly relevant for the occurrence of large‐scale precipitation extremes in Central Europe (Messmer et al., 2015), with up to 45% of these cyclones being associated with heavy precipitation in the Czech Republic and Eastern Austria (H18). At the same time, Vb cyclones are rather rare, as only 5% of all Central European cyclones can be attributed to this track type.

The temporal evolution of Vb cyclone occurrence has not been investigated so far. Observed trends of the total number of cyclones developing over the WM Sea may shed some light on the frequency of Vb cyclones. Lionello et al. (2016) examined WM cyclone tracks over the period 1979–2008 on an annual basis, but did not find any significant trends. Maheras et al. (2001) found a decrease in frequency of −4% per decade during 1958–1999, and a similar decrease was detected by Bartholy et al. (2009) which they attributed to the winter/spring season.

Another important, unresolved issue is whether Vb cyclones tend to occur in clusters, that is, whether their arrival rate in some periods is significantly higher than the rate expected for a random Poisson point process (Cox & Isham, 1980). The occurrence of clusters would not only raise the question of their causal mechanism, but would also be of high practical relevance for flooding because of the accumulation of soil moisture by repetitive precipitation events (Grillakis et al., 2016). For example, a number of floods in the Isar catchment in southern Germany were exacerbated by a sequence of two Vb cyclones (Stahl & Hofstätter, 2018). Additionally, gearing flood risk management strategies toward flood‐rich periods is of enormous practical importance (Hall et al., 2014).

Serial clustering of cyclones is well known to occur over Western Europe (Mailier et al., 2006; Pinto et al., 2013; Vitolo et al., 2009) which is triggered by a persistent, zonally orientated, and extended eddy‐driven polar jet stream over the North Atlantic (Pinto et al., 2014). A persistent jet stream, which varies little in latitude, is typically associated with a strong jet (high wind speeds) located around 45°N (Woollings et al., 2010, 2018). In contrast, if the jet stream is weak and shifted toward the South, that is, the location of the WM, cyclogenesis and even clustering in the WM might be enhanced. The southern position of the jet stream is largely determined by a corresponding negative phase of the Northern Atlantic Oscillation (NAO; Hurrell, 1995; Wallace & Gutzler, 1981; Woollings & Blackburn, 2012), which is usually associated with an increased frequency of high latitude blocking over Greenland or Northern Europe (Woollings et al., 2008). Indeed, during NAO^−^ conditions, the number of cyclone tracks in the WM is about +20% larger than during NAO^+^ (Nissen et al., 2010, 2014). Overall, these findings suggest that the occurrence of Vb cyclones could be connected to a specific state of the large‐scale atmospheric circulation (LAC). Conversely, Vb cyclones are one of the strongest European cyclone types (Hofstätter et al., 2016, H16 hereafter), so one would also expect a considerable effect of Vb events on the large‐scale circulation. If a characteristic atmospheric flow state exists favoring the propagation of cyclones on track Vb or even the genesis of Vb cyclones in the WM, this could also point toward a plausible, yet unexplored, mechanism explaining clusters of Vb cyclones. A link between cyclogenesis in the WM and the upper level dynamics interacting with major orography in a low‐level baroclinic environment may contribute to these processes (e.g., Maheras et al., 2002; Trigo et al., 2002).

The aim of this paper is to understand the occurrence of Vb cyclones over time, and the atmospheric processes associated with them, by investigating daily time series of atmospheric reanalysis data for the period 1959 to 2015. Specifically, the paper addresses the following questions: (1) Have Vb cyclones become more frequent in recent decades and does their rate of occurrence reveal characteristic variations over time; (2) do Vb cyclones tend to occur in clusters or do they arrive fully randomly; (3) do clusters, or hiatus periods, correspond with specific phases of the dominant modes of the LAC; and (4) does such a relationship, if it exists, suggest a plausible mechanism for a possible self‐exciting process of Vb occurrence? Self‐exciting means that the current rate depends on the history of the process (Hawkes, 1971), meaning that one Vb event increases the probability of a successive one as compared to a certain background rate.

The paper is organized as follows: The data and methods used are explained in section 2, followed by an analysis of the temporal features of Vb cyclone occurrence and its relationship to the LAC in section 3. Section 4 provides the conclusions.

## 2 Data and Methods

The cyclone tracks used in this study are identified by the detection and tracking algorithm of Hofstätter et al. (2016, 2018) which is based on Murray and Simmonds (1991) and Simmonds et al. (1999) and considers both open and closed systems (Pinto et al., 2005). The algorithm consists of four steps: (i) identification of cyclones at time t, (ii) prediction of a subsequent cyclone position at time t + 1, (iii) association of cyclones between times t and t + 1 by scoring the difference between the predicted and actual cyclone position(s), and (iv) removal of spurious tracks. Cyclones are identified by detecting local minima of geopotential height at the level of 700 hPa (Z700) or air pressure at mean sea level (SLP). For closed cyclones all four neighboring derivatives must be positive whereas for open systems one derivative is less or equal zero. In case of the latter only the most intense point is considered along the same trough axis, using geostrophic relative vorticity as intensity measure. For avoiding spurious cases, the data are filtered by a spatial low‐pass filter first of all and weak/shallow cyclones rejected subsequently. In a final step resulting tracks are screened to remove implausible cases, such as short living and slow or erratically moving systems. As an important strength of the current tracking scheme, splitting and merging of cyclones is permitted, consequently all branches of a complex track are retained before classifying individual tracks. More detailed information on the tracking procedure and the specific parameter values chosen is described in Hofstätter et al. (2016).

The tracking procedure is applied within a domain ranging from 40°W to 50°E at 65°N and 20°W to 4°E at 3°N for the years 1959–2015. The tracking analysis and all atmospheric fields are based on the JRA‐55 reanalysis (Harada et al., 2016; Japanese Meteorological Agency, 2013; Kobayashi et al., 2015) retrieved from the Research Data Archive at the National Center for Atmospheric Research at 1.25° spatial and 6‐hr temporal resolutions.

**Figure 1:**
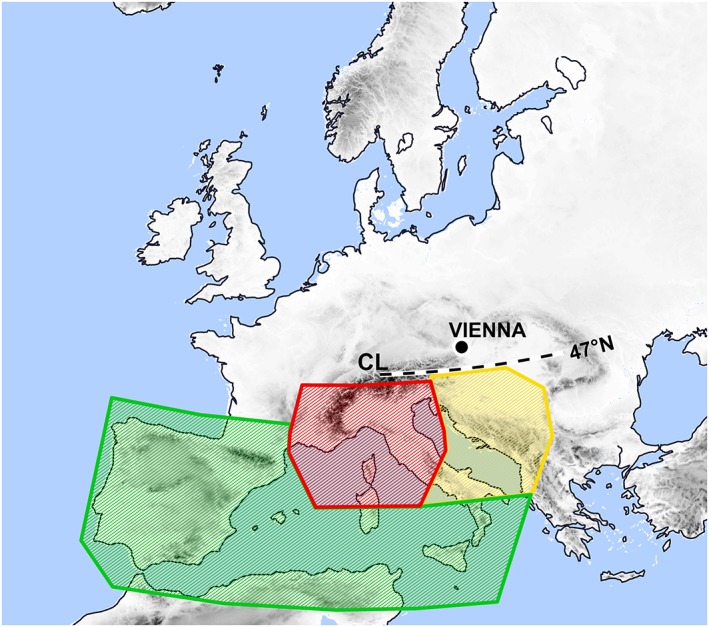
Study region with four source regions for defining Vb subtypes GoG (red), DiN (yellow), IbA (green), and OuT (all others; Table 1). Cyclone tracks are identified as Vb if they cross the black dashed line (CL) from the south. CL = Crossing Line.

Recent studies (H18; Messmer et al., 2015; Nissen et al., 2013) have used different source and/or target regions for identifying Vb cyclone tracks which hampers a comparison of results. A less restrictive definition than the one of H16 and H18 is therefore used here. All cyclones that propagate northward and cross a line at 47°N between 12 and 22°E (“Crossing Line, CL,” in Figure 1) are identified as Vb tracks (Vb‐All). The arrival time d_0_ is defined as the point in time when the cyclone crosses that line, which is estimated by linear interpolation between the track positions bracketing the line.

Tracks are first identified at two atmospheric levels, Z700 and SLP, independently and subsequently considered jointly, in order to include Vb cyclones appearing at Z700 exclusively as well. In order to avoid redundancies, Z700 tracks are considered only if they are deemed to be independent of all other SLP tracks. Two conditions are used to identify whether Z700 and SLP tracks refer to the same system: (i) the overlap lasts for at least 18 hr; (ii) the median and the twentieth percentile of the horizontal distances between the track positions at the same time are less than 1,000 and 700 km, respectively. This criterion is based on the observation that, at their peak, cyclones at SLP are typically 300 km ahead of the associated Z500 trough (Lim & Simmonds, 2007); (iii) Z700 tracks are only considered if they are separated by at least 24 hr when passing the CL line; otherwise the cyclone with a lower value of geostrophic relative vorticity at d_0_ is rejected. By this screening, a total of 100 Vb events (30%) are disregarded at Z700. If one increases (decreases) the threshold distances by 30%, the detection rate remains very similar with 104 (97) events, so the rejection rate appears robust to the choice of the threshold distances.

The remaining cyclones are classified into four Vb subtypes (Table 1), depending on the region the cyclone develops (Figure 1). The classification is motivated by the existence of distinct cyclogenesis regions in the WM Sea with contrasting cyclogenesis mechanisms in different seasons (Campins et al., 2010; Flaounas et al., 2018; Lionello et al., 2016; Trigo et al., 1999; Trigo et al., 2002). These regions are the Gulf of Genoa/Ligurian Sea and Northern Italy, the Adriatic Sea and Dinaric Alps, the Iberian Peninsula and North African Coast (Figure 1 and Table 1).

**Table 1:** Four Subtypes of Vb Cyclones, Differentiated by Their Source Region

Sub	Acronym	Color	Description of source region
1	GoG	Red	Gulf of Genoa/Ligurian Sea and Northern Italy
2	DiN	Yellow	Adriatic Sea and Dinaric Alps
3	IbA	Green	Iberian peninsula and North African Coast
4	OuT	/	All other Vb cyclones that are not of subtype 1–3

Following the work of Mailier et al. (2006), Vitolo et al. (2009), Pinto et al. (2013, 2016), and Walz et al. (2018), the arrival of a cyclone at a particular location is considered as a realization of a Poisson point process (Cox & Isham, 1980). The probability of *n* arrivals in time interval ∆t is given by

(1) $$P_{\left(N=n\right)}=\frac{\mu^{n}}{n^{!}}\cdot e^{-\mu }\;\left(n=0,1,2,\ldots \right),$$

where *N* is the discrete random variable of the arrivals, μ is the expected number of arrivals in ∆t with μ = λ · ∆t, and λ the arrival rate. The ratio of the variance Var(*N*) and the mean E(*N*) is used to estimate the dispersion measure *φ* (Mailier et al., 2006) as

(2) $$\varphi =\frac{\mathrm{Var}\left(N\right)}{E\left(N\right)}-1,$$

For a homogeneous Poisson point process (λ does not change over time) *φ* = 0, if *φ* > 0 (overdispersion) cyclones arrive more clustered, whereas if *φ* < 0 (underdispersion) they arrive more regularly than if the interarrival times were independent. The inter arrival times *T* are exponentially distributed with density

(3) $$f_{\left(T\right)}=\lambda \cdot e^{-\mathrm{\lambda T}}\;\left(\lambda >0;T>0\right)$$

and mean λ^−1^. In order to test if the observed interarrival times are drawn from an exponential distribution, the Anderson Darling Test is applied (Stephens, 1974).

Clusters are usually defined as distinct periods with a markedly higher arrival rate than the average rate λ_0_(Pinto et al., 2014). In this study a cluster is identified if the daily cyclone count C over a moving window of 180 days (6 months) exceeds twice the expected number for this period length, so if C^±3m^ > 2 · μ_6*m*_. So for every single day the moving average is checked if exceeding the threshold. Complementing clusters on a shorter time scale are identified over a window of 90 days (3 months), that is, C^±1.5m^ > 2 · μ_3*m*_, and considered if continuing a precedent 6 m‐cluster. The selected thresholds correspond approximately to the 5% exceedance probability. Similarly, a hiatus is defined as a period of days with a much smaller number of Vb events, that is, if the 6‐month cyclone count drops below half the expected number, C^±3m^ < 0.5 · μ_6*m*_. This procedure results in a total of 16% of all days between 1959 and 2015 identified as clusters and 35% of all days identified as hiatuses.

The time‐depending arrival rate is estimated by a Gaussian kernel (width = 5 years) with percentile‐t confidence intervals 1‐2α = 0.90% calculated by ordinary bootstrapping (K = 8,000 resamples) with replacement (Cowling et al., 1996; Mudelsee et al., 2003, 2004; Mudelsee, 2014). The seasonal change of Vb arrivals is analyzed by the Gasser and Müller (1979, 1984) kernel regression with a parabolic kernel function (width h = 10 years, Mudelsee et al., 2012). The standard error bands are constructed by moving blockwise‐bootstrapping using an adopted block length considering serial dependence (Mudelsee, 2014, his equation 3.28) as estimated through persistence time *τ* (Mudelsee, 2002).

Most of the analysis in this study is based on daily time series but, at some instances, results are aggregated over seasons, either for winter (December‐January‐February, DJF), spring (March‐April‐May), summer (June‐July‐August, JJA), and autumn (September‐October‐November) or for the winter and summer half years (November to April and May to October). If not stated otherwise, summer and winter refers to the half years.

To investigate the relationship between Vb cyclone occurrence and the LAC, time series (indices) of northern hemispheric teleconnection patterns are used in this study. These are primarily the NAO and the Arctic Oscillation (AO) and secondarily the East Atlantic/Western Russia Pattern, the Scandinavian Pattern, the Polar Eurasia Pattern, the Eastern Atlantic Pattern, the East Pacific/North Pacific Pattern, and the Pacific/North American Pattern (Barnston & Livezey, 1987; Feldstein & Franzke, 2017). As the most prominent pattern, the NAO (Hurrell, 2001) correlates with the European climate and explains precipitation variability over Central and Western Europe (e.g., Hurrell et al., 2003). All indices are provided by the Climate Prediction Center (CPC) of the National Oceanic and Atmospheric Administration (http://www.cpc.ncep.noaa.gov/data/teledoc/telecontents.shtml). All CPC teleconnection patterns refer to 500 hPa GPH anomalies with the exception of AO which refers to 1,000 hPa. For comparison with the CPC NAO index (denoted as NAO_c), an alternative index (denoted as NAO_n) from National Center for Atmospheric Research's Climate Analysis Section based on the leading mode of SLP anomalies is used (Hurrell et al., 2003).

For a number of analyses, confidence intervals are constructed by ordinary bootstrapping (with replacement) unless stated otherwise.

## 3 Results and Discussion

### 3.1 A Brief Climatology

For the period 1959–2015, a total of 557 Vb cyclones are identified, averaging about 9.8 cyclones per year (Table 2) and with a corresponding quartile deviation (pct_75_‐pct_25_) of 3.5 year^−1^. The number of Vb cyclones at SLP (6.1 year^−1^) and at Z700 (3.7 year^−1^) is higher than that of Hofstätter et al. (2016) with 4.1 year^−1^ and 3.1 year^−1^, and also higher than that of Messmer et al. (2015) with 2.5 year^−1^ at Z850 and Nissen et al. (2013) with just 1.2 year^−1^ at SLP. The latter two studies used a very stringent definition for Vb tracks (see their Figure 2), and they only used a single pressure level for track detection. In the current study the definition is less restrictive, classifying all cyclones propagating northward at 47°N between 12 and 22°E as Vb, either at SLP or at Z700. As a drawback, region DiN is contiguous to the CL potentially leading to ambiguous cases when cyclones jump across CL temporarily. In this study 17 DiN cyclones start close to the CL (>45°N) but only three of them do not propagate beyond 48°N subsequently, so these are not expected to impact the results.

**Table 2:** Vb Cyclone Climatology With the Total Number of Cyclones for the Period 1959–2015, the Average Number per Year μ, the Percentages for the Summer and Winter Seasons, and the Fraction of Cyclones Identified From SLP Relative to the Total Number of Vb Cyclones (JRA‐55: SLP and Z700)

Vb‐cyclones	ALL	GoG	DiN	IbA	OuT
Total number	557	257	98	145	57
μ (year^−1^)	9.77	4.51	1.72	2.54	1.00
May‐Oct (%)	43.8	48.3	46.9	35.9	38.6
Sept‐Apr (%)	56.2	51.7	53.1	64.1	61.4
Fraction of SLP cyclones	0.62	0.57	0.64	0.77	0.44

*Note*. SLP = sea level pressure.

**Figure 2:**
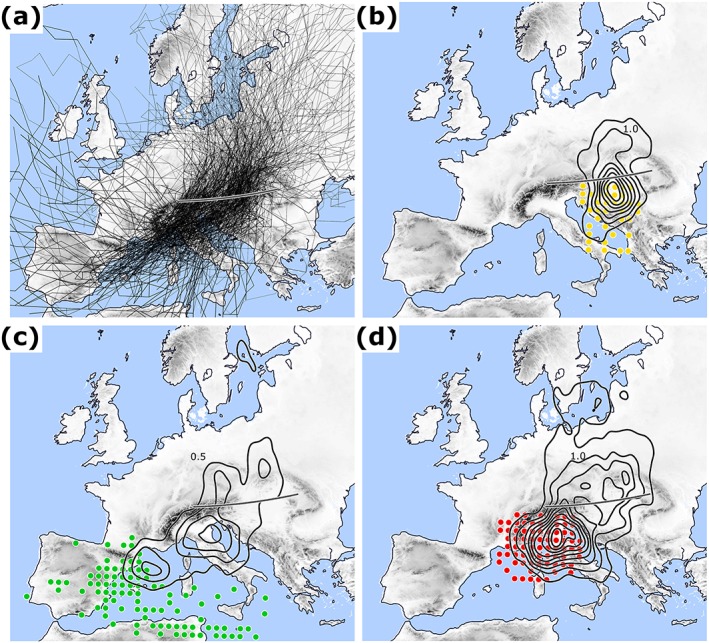
(a) Individual tracks for all 557 Vb cyclones (JRA‐55, 1959–2015: composite of sea level pressure and Z700). (b–d) Point of first detection for subtypes DiN (b), IbA (c), and GoG (d) shown as color coded dots according to the subregions of Figure 1. Black contours indicate the location where the cyclones reach their lifetime minimum of air pressure or geopotential height (cyclone count at grid points with 0.5 intervals). Subtype OuT is omitted in (b–d) because of their low cyclone number.

In terms of subtypes, Vb‐GoG is most frequent (4.5 year^−1^ or 46%), followed by Vb‐IbA (2.5 year^−1^ or 26%), Vb‐DiN (1.7 year^−1^ or 18%), and Vb‐OuT (1.0 year^−1^ or 10%). The Gulf of Genoa is not only the most active cyclogenesis region in the WM (Campins et al., 2010; Reale and Lionello, 2013), but also appears to be the most relevant source region of Vb cyclones. IbA and OuT subtypes are more frequent in winter; the other subtypes are balanced between winter and summer. Figure 2a shows the individual cyclone tracks for Vb‐ALL. Figures 2b–2d show the corresponding starting points (first detection) ‐d for subtypes DiN, IbA, and GoG. In addition, black contour lines mark the regions where the cyclones reach their minimum of air pressure or geopotential height, calculated as cyclone count per grid point. GoG and DiN cyclones reach their minimum very close to their genesis region and just before crossing the CL, whereas for IbA cyclones this region is more diffuse and ranges from the Balearic Island to Central/Eastern Europe. This indicates that the genesis of GoG and DiN cyclones is more strictly bound to specific geographical or environmental conditions than that of IbA.

### 3.2 Occurrence Rate and Temporal Changes

Vb cyclones are rather rare with only 5% of all Central European cyclones assigned to this track type (H18). Despite its rarity, 22 of the 50 largest precipitation events in the Czech Republic and eastern Austria (1959–2015) have been attributed to Vb cyclones (H18). This implies that the probability of heavy precipitation associated with Vb cyclones at a given location is high. The arrival of Vb cyclones is therefore regarded as an extreme event in the following.

As shown in Table S1, the probability of occurrence of Vb (ALL) cyclones has decreased (*U* < 0) between 1959 and 2015 (*p* = 0.05) which can be mainly attributed to IbA winter cyclones, using the Cox‐Lewis test for a monotonic trend (Cox & Lewis, 1966, p. 47). If one disregards IbA‐Winter cyclones (second column in Table S1) the probability still decreases, however without significance (*p* = 0.19). The square root‐transformed and linearly detrended interarrival times are checked for serial dependence which gives lag‐1 correlation coefficients between *R*_1_ = −0.18 (OuT) and +0.13 (DiN), and *R*_1_ = +0.05 for ALL (Table S1c), indicating weak autocorrelation.

**Figure 3:**
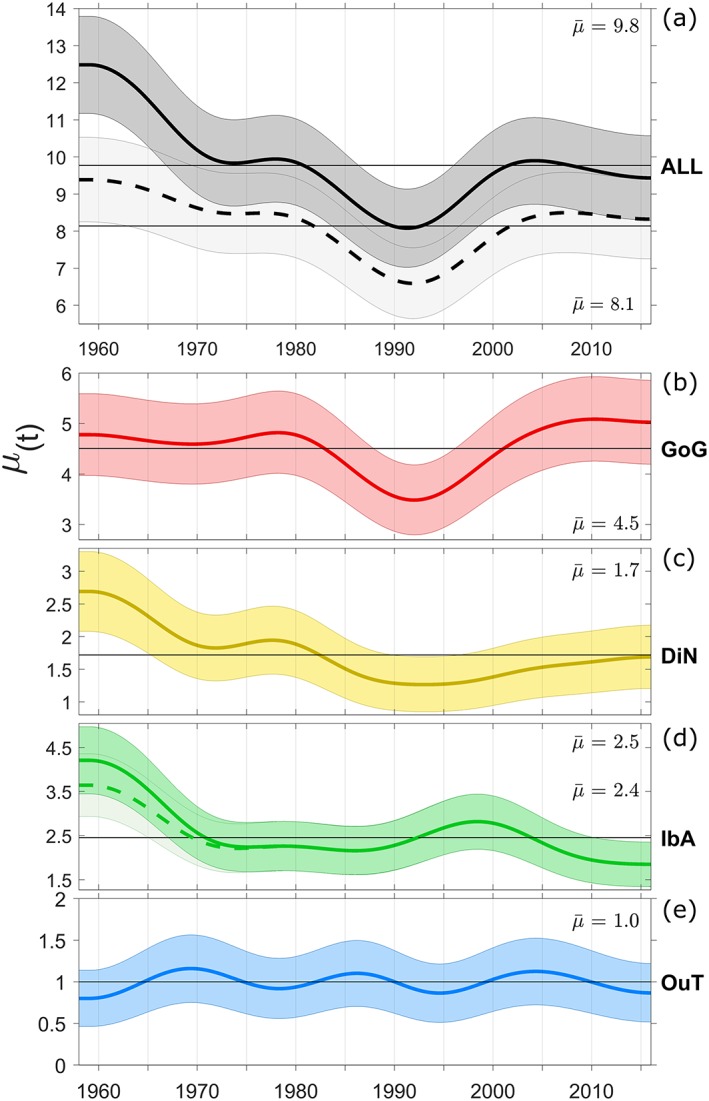
Vb cyclone frequency. (a) all Vb cyclones, and subtypes (b) GoG, (c) DiN, (d) Iba, (e) OuT. Occurrence rate (year^−1^) estimated by a Gaussian kernel of bandwidth h = 5 years with percentile‐t CI 1–2α = 0.90 indicated as shaded areas. The top panel gives the occurrence rate for all Vb‐cyclones (solid line, dark gray CI) and for all cyclones excluding IbA‐winter (broken line, light gray CI). The dashed green line for IbA indicates the occurrence rate if five out of nine cyclones in winter 1962/1963 are disregarded. The stationary occurrence rate $\bar{\mu }$ (year^−1^) is indicated on the right. CI = confidence interval.

Figure 3 shows the occurrence rate of Vb cyclones for the period 1959–2015 estimated by a Gaussian kernel using a bandwidth of h = 5 years (Mudelsee, 2014; Mudelsee et al., 2004). The black horizontal line indicates the average rate $\bar{\mu }$ with the numbers given in each panel. Clearly, Vb occurrence is time dependent with distinct decadal variations.

For all cyclones (Vb‐All, Figure 3a) the rate is highest in the 1960s but does not show a clear increase or decrease between 1975 and 2015. Since the 1960s near surface air temperatures have increased by 0.9° over the Northern Hemisphere (Jones et al., 2012; Morice et al., 2012; Osborn & Jones, 2014). At the same time the meridional temperature gradient and baroclinity over the Northern Atlantic has weakened between October and March from 1979 to 2015, suggesting a decrease of cyclone frequency and/or intensity there as well (Wang et al., 2017). In line with our findings, the frequency of Mediterranean cyclones including Vb did not change significantly either (Lionello et al., 2016), based on a multitracking analysis using ERA‐Interim data for 1979–2008. Only when considering the entire period 1959–2015, the rate does decrease slightly by −4% per decade in this study, which is identical with the changes of WM cyclones for 1958–1999 identified by Maheras et al. (2001).

The frequency of Vb‐ALL is at a very low level of 8 .2 year^−1^ during the early 1990s. This hiatus phase is denoted as H7 and investigated in more detail later in this paper. In the early 1960s the occurrence rate is larger than 12 year^−1^, which can be attributed to the high number of IbA winter cyclones, as illustrated by the dashed black line in Figure 3a for which IbA‐winter cyclones have been excluded. During this phase, IbA (Figure 3d) exhibits a substantially higher rate of >3 .5 year^−1^ than after 1965. However, when removing every other IbA cyclone (five out of nine) in winter 1962/1963 as a sensitivity experiment, the rate drops remarkably (Figure 3d, dashed line). During this specific winter, a total of nine Vb‐IbA cyclones have occurred compared to the long‐term average of 1.6 per winter. The winter 1962/1963 was a severely cold one in Northern and Central Europe and was associated with a sustained negative NAO regime (Greatbatch et al., 2015). DiN exhibits a similarly high rate before 1970, followed by a rather stable occurrence rate afterward.

**Figure 4:**
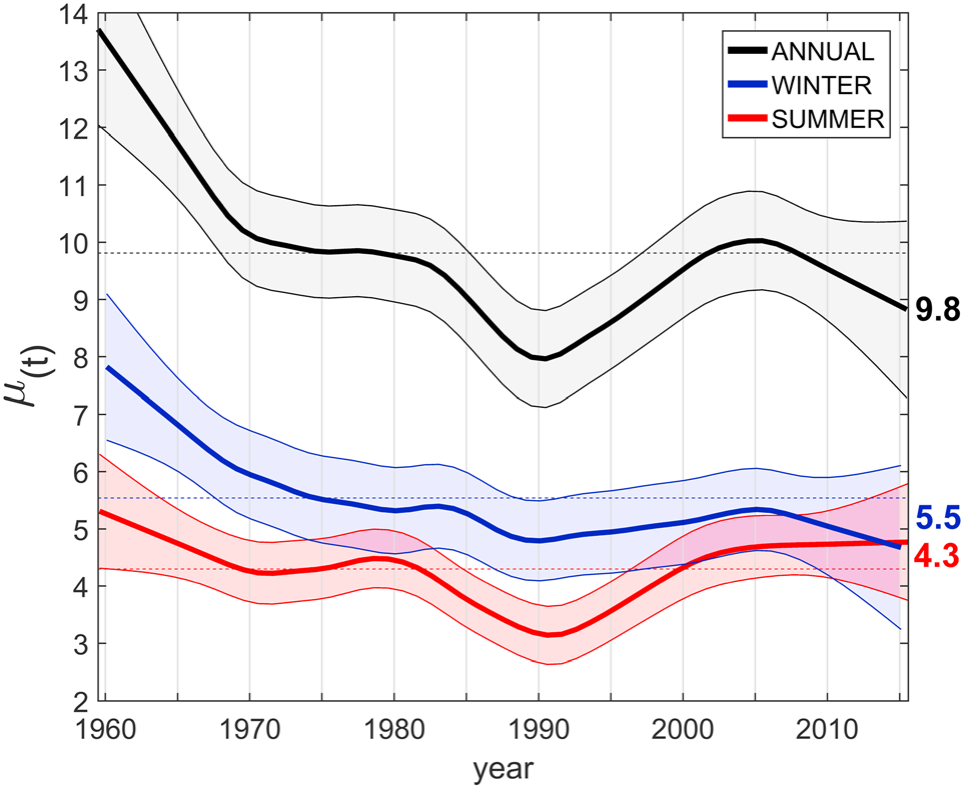
Seasonal and annual occurrence rate (year^−1^) of Vb cyclones. Gasser‐Müller kernel regression estimation with a parabolic kernel function and 1‐σ error bands from K moving block bootstrap experiments (K = 8,000) using a predefined persistence time τ = 0.3 (winter), τ = 0.1 (summer), and τ = 0.15 (annual).

Vb winter cyclones were significantly more frequent before 1967 than later and are now at their lowest rate since 1959 (Figure 4). The rate in winter has decreased significantly by about 40% from 8.03 year^−1^ (±1.40) in 1959 to 4.68 year^−1^ (±1.44) in 2015, in line with Figure 3d. A similar decrease of WM cyclones in winter/spring was detected by Bartholy et al. (2009). The summer occurrence rate in this study is generally close to its long‐term average and is at 4.3 year^−1^ (±1.0) in 2015. The significantly lower rate during the early 1990s (H7) not only emerges from Vb‐GoG (Figures 3a and 3b) but it is also more prominent in the summer (Figure 4).

### 3.3 Dispersion of Vb Cyclones

So far we have shown that Vb cyclones occur at a time‐varying rate with largely independent interarrival times. For a Poisson point process, interarrival times T should be exponentially distributed which is examined in Figure S1. Additionally, the Anderson‐Darling test is used to test whether the sample comes from an exponential distribution (H0: “interarrival times are iid exponentials”). Figure S1 and the tests suggest that the interarrival times of GoG and IbA cyclones are not exponentially distributed which may be related to the time‐varying rates λ_(t)_ (Figures 3 and 4) and the enhanced frequency of short interarrival times (Figure S1, GoG, IbA). This is a clear indication of overdispersion, implying clustering of the arrivals. This finding raises the question of the driving mechanisms of clustering, and the relevance for flooding due to the accumulation of soil moisture in the same catchments (Grillakis et al., 2016; Komma et al., 2008). In order to more explicitly test for clustering of the cyclone arrivals, the dispersion measure *φ* (Mailier et al., 2006) is estimated for the annually and seasonally aggregated number of cyclones.

As shown in Table 3, Vb cyclones indeed occur in clusters, which can be mainly attributed to the GoG and IbA subtypes in accordance with Figure S1. Subtype DiN cyclones occur more regularly than a random Poisson process. On an annual basis (Table 3b), *φ* indicates clustering for all types. Seasonally (Table 3c), clustering is much more pronounced in winter (*φ* = 0.78) which is mainly due to IbA (*φ* = 0.61). In contrast, clustering of GoG cyclones which develop on the southern Alpine lee side is particularly strong in summer (*φ* = 0.71). This finding points toward differences in the formation processes of clusters in different seasons. The mean interarrival times $\bar{T}$ (Table 3, a), as derived from the inverse of the rate parameter of the exponential (equation (3)) distributions, are about 37.4 days for all Vb cyclones in correspondence to the rate of 9.77 events per year of Table 2.

**Table 3:** Dispersion Statistics of the Vb Cyclone Arrivals, With φ > 0 Indicate Clustering of Cyclone Arrivals and φ < 0 Indicate Regularity

	Clustering indicator	ALL	GoG	DiN	IbA	OuT
(a)	$\bar{T}$ (days)	37.43	81.0	212.5	143.6	365.3
(b)	*φ* (annual)	+0.69	+0.33	−0.09	+0.56	+0.11
(c)	*φ* (summer)	+0.25	+0.71	−0.10	−0.09	+0.00
	*φ* (winter)	+0.78	+0.05	−0.13	+0.61	+0.21

The higher arrival rates of Vb cyclones during clustering periods could be caused (i) by a sustained large‐scale atmospheric state which forces a repetitive development of Vb cyclones in the WM and/or (ii) by a self‐exciting process in which the Vb event itself reinforces the LAC into a state that favors the development of a successive Vb cyclone (positive feedback).

**Figure 5:**
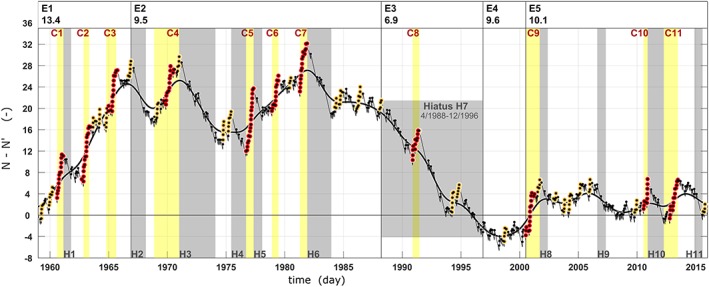
Daily arrivals of Vb‐All cyclones from 1 January 1959 (t = 1) to 31 December 2015 (t = 20820) shown as the cumulative number of cyclones observed (N) minus expected (N′) $=\sum_{t=1}^{20820}\left(N_{\left(t\right)}-\lambda_{0}\cdot t\right)$ assuming a constant rate λ_0_ = 0.0268 d^−1^ for the latter. Black smooth curve indicates running average using a 5 year^−1^ Gaussian low‐pass filter. Individual Vb events (n = 557) are shown as black dots. Clustered events are indicated by red dots. Cluster periods (C) and hiatus periods (H) are indicated by yellow and gray shading, respectively. The observation period has been partitioned into five main episodes E1 to E5 with average arrival rates (per year) given at the top.

Figure 5 identifies cluster and hiatus periods for Vb‐ALL by plotting the sample cumulative arrivals minus their average over the period ( $\bar{\mu }$ = 9.77 year^−1^). The breakpoints separating the major episodes E1 to E5 are detected by a break regression model (Mudelsee, 2009). The highest arrival rate of 13.4 year^−1^ occurs between January 1959 and December 1966 (episode E1), the lowest arrival rate of 6.9 year^−1^ between April 1988 and December 1996 (hiatus H7). During other periods, the rate is close to the long‐term average. In total there are 11 cluster and 11 hiatus periods between 1959 and 2016 (Table 4).

**Table 4:** Vb Clustering (C) and Hiatus (H) Periods 1959–2016, With Beginning and End Dates, Duration, Mean Interarrival Times T, Number of Arrivals, and the Ratio (fR) of Arrival Rates and the Mean Rate of 9.77 year^−1^

C/H	Beginning	End	Duration (months)	T (days)	Number of arrivals	fR (%)
C1	1960 08 11	1961 02 20	6.33	14.9	14	271
C2	1962 11 08	1963 05 04	5.80	11.8	16	338
C3	1964 10 13	1965 08 24	10.33	16.6	20	237
C4	1968 11 17	1971 01 03	25.48	23.5	34	164
C5	1976 09 13	1977 05 07	7.74	13.1	19	301
C6	1978 11 26	1979 06 23	6.85	17.4	13	233
C7	1981 04 16	1981 11 28	7.41	15.1	16	265
C8	1990 11 22	1991 06 17	6.79	18.8	12	217
C9	2000 07 09	2001 09 17	14.26	19.8	23	198
C10	2010 07 22	2010 11 29	4.26	14.4	10	288
C11	2012 04 11	2013 06 25	14.43	23.2	20	170
H1	1961 02 20	1961 10 18	7.87	60.0	4	62
H2	1966 11 24	1968 03 02	15.21	154.7	3	24
H3	1971 01 03	1974 02 03	36.95	75.1	15	50
H4	1975 06 09	1976 09 13	15.15	92.4	5	40
H5	1977 05 07	1978 01 28	8.72	133.0	2	28
H6	1981 11 28	1983 12 19	24.62	93.9	8	40
H7	1988 03 21	1996 11 17	103.71	52.7	60	71
H8	2001 09 17	2002 05 26	8.23	83.7	3	45
H9	2006 08 13	2007 05 05	8.69	88.3	3	42
H10	2010 11 29	2012 04 11	16.36	99.8	5	37
H11	2014 11 19	2015 08 01	8.36	127.5	2	29

*Note*. Dates are in the format year month day.

Figure 5 clearly shows the existence of well‐separated and distinct clusters (C1–C11) with a relatively high arrival rate of $\bar{\mu }$ = 21.5 year^−1^, and a low average interarrival time of $\bar{T}$ = 17.0 days on average, compared to $\bar{\mu }$ = 7.5 year^−1^ and $\bar{T}$ = 48.5 days outside the clusters. During the hiatus periods the mean interarrival time is particularly long with $\bar{T}$ = 70.4 days and the rate is only $\bar{\mu }$ = 5.2 year^−1^, suggesting that the cyclone clusters exhibit an average rate about four times that of the hiatuses. It is interesting to note that cyclone clusters over the Northern Atlantic Ocean occur at a much higher rate of about 1 day^−1^ and with much shorter cluster duration of just 6 days on average for the main winter (Pinto et al., 2014). Obviously, the formation processes of cyclone clusters over the Northern Atlantic are different from those in the WM. Nevertheless, the ratio of the number of clustering days to all days over the Northern Atlantic of 13% is very similar to the one found here for the WM (16%).

### 3.4 LAC

In order to better understand the relationship between the LAC and Vb cyclone arrival rates, selected leading modes of atmospheric variability and arrival rates is investigated. The main questions are the following: (i) does the NAO or AO favor the development of clusters or hiatus periods and can therefore explain long‐term changes in arrival rates, (ii) does the LAC show any response in the event of Vb, and (iii) does the evolution of the LAC during Vb events support the hypothesis of a positive feedback? The last question is particularly intriguing as cyclogenesis strongly depends on a sufficient upper level forcing which is usually much stronger in the winter than in summer while Vb cyclones are just as frequent in the winter as in summer (Table 1) and Vb summer cyclones, on average, are equally strong as the winter ones (H16). In the WM, upper level troughs are usually affected by major mountain orography inducing lee‐cyclogenesis in a low‐level baroclinic environment (Aebischer & Schär, 1998; Egger, 1988; Pichler et al., 1990; Trigo et al., 2002), suggesting that either low‐level baroclinic forcing compensates a weakened upper level dynamics during summer or Vb occurrence rates are modulated by different states of the LAC in different seasons.

#### 3.4.1 Long‐Term Variations in Arrival Rates

Greatbatch et al. (2015) found an exceptionally high number of nine Vb‐IbA events during the winter 1962/1963 when a sustained negative phase of the winter NAO was observed, and Nissen et al. (2010) found Mediterranean cyclones to occur about 20% more frequently during negative NAO phases. Negative NAO conditions are typically related to a weakened, more variable, and southward shifted, eddy‐driven jet stream (e.g., Woollings et al., 2008, 2010), hence steering cyclogenesis and cyclone tracks toward more southern latitudes than usual. The mean monthly state of selected teleconnection indices ( ${\bar{\mathrm{TCI}}}_{k}$) is shown in Figure S2. Very clearly the AO, NAO, and East Atlantic/Western Russia patterns are in a negative state during months when Vb cyclones occurred ( ${\bar{\mathrm{TCI}}}_{k}<0$) but are in a more positive state otherwise ( ${\bar{\mathrm{TCI}}}_{0}>0$). These indices are more strongly negative with an increasing number *k* of Vb events per month. A similar, however reverse relationship can be seen for the Scandinavian pattern. For the other modes such a clear tendency cannot be seen.

**Figure 6:**
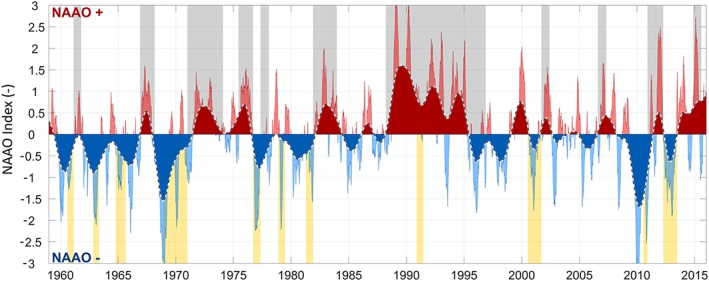
Time series of the combined standardized daily NAO/AO index for 1959–2015. Dark blue/red: 3‐year Gaussian‐low pass filtered; light blue/red: 6‐month Gaussian‐low pass filtered index. Yellow shaded areas indicate cyclone clusters, gray shaded areas hiatuses as in Figure 5.

Figure 6 shows the composite time series of the averaged NAO/AO index (denoted at NAAO in the following) together with the clustering (yellow) and hiatus (gray) phases. The NAAO is calculated from the individual standardized and deseasonalized time series as (NAO + AO) / 2. The combined NAAO index is averaged (running mean) over 3 years (dark red and blue) and 6 months (pale red and blue). The most prominent hiatus phase H7 between April 1988 and December 1996, which has been attributed more strongly to the summers (Figure 4) occurs during a sustained and strongly positive phase of the NAAO (also see, e.g., Delworth et al., 2016). The high annual arrival rate of Vb cyclones found before 1971 (Figure 3), which has been attributed to the winters (Tables 3 and 4 and Figure 4), goes hand in hand with a predominantly negative phase of the NAAO. This suggests that the rate of Vb cyclones may be elevated under negative NAAO conditions in the winter and suppressed under positive NAAO conditions in the summer.

**Figure 7:**
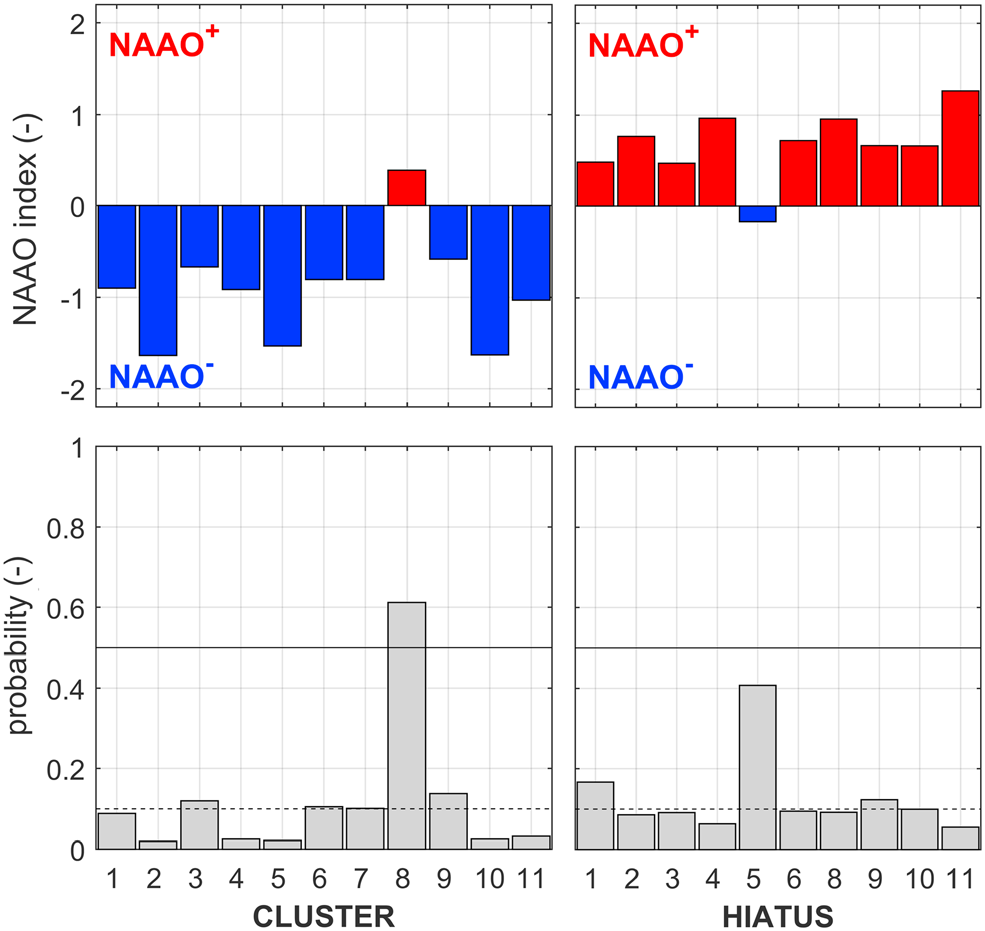
Mean state of the NAAO for the cluster and hiatus periods as an average over their duration (top) as well as the probability (bottom) of observing an even stronger negative (positive) phase of the NAAO, given the duration d of a particular cluster (hiatus). Dashed lines indicate significance (p = 0.10). Hiatus H7 has been omitted because of its exceptionally long duration.

Almost all the hiatus phases (gray) and most cyclone clusters (yellow) in Figure 6 coincide with positive and negative phases of the NAO, respectively. For a more quantitative assessment, the mean states of the NAAO index during clusters and hiatuses are evaluated in Figure 7, top. At the bottom of Figure 7 the probability of observing an even stronger negative (positive) phase of the NAAO is shown, given the duration *d* (unit days) of the particular cluster (hiatus) under consideration. The probabilities are calculated by drawing 10^6^ ordinary bootstrap samples with replacement, whereas the period under consideration is always omitted. Significance is indicated by a probability of 0.10, shown as dashed lines in Figure 7, bottom.

For all clusters, except C8, the average NAAO is clearly negative with a rather low probability (less than 0.20) of observing an even more negative phase of the NAAO for the given period length. For six clusters the state of the NAAO is significantly (*p* < 0.10) more negative as compared to random occurrence. A similar conclusion can be drawn for 9 of the 10 hiatus periods with 6 of them being significantly more positive than random occurrence. Only hiatus H5 occurs during a slightly negative phase of the NAAO. This general relationship between Vb occurrence and the NAAO, however, does not strictly apply to each cluster or hiatus. From inspecting individual Vb events of C8 (not shown), we find that some Vb cyclones occur during a positive NAAO phase. In other instances, the AO or NAO drops as expected, but remains positive most of the remaining time, so is not captured well by the 6‐month running average of the NAAO.

This finding confirms that, on the medium term (between 6 months and 3 years), hiatuses almost always occur during positive phases of the NAAO, and cyclone clusters during negative phases. This is in line with the observation of Nissen et al. (2010) that the number of cyclones in the WM is about 20% lower during NAO^−^ phases than during NAO^+^ phases. Similarly, strong WM cyclones were found to be correlated (*R* = 0.50) with a NAO^−^ type pattern in winter (DJF), whereas this relationship was lost in summer (JJA; Raible, 2007). Although our results show a very close relation between the NAAO and Vb cyclone occurrence, it is important to understand whether this specifically applies to Vb‐cyclones or to all WM cyclones, as the number of the latter is roughly eight times higher. To this end, the fraction of the mean number of cyclones between (i) clusters and hiatuses and (ii) NAAO^−^ and NAAO^+^ (3‐year Gaussian LP filtered) is compared. In the following WM_ALL_ refers to all cyclones developing either in the IbA, GoG, or DiN region (Figure 1) and Vb_ALL_ to those WM_ALL_ cyclones subsequently following the track Vb.

The ratio (i) is 1.39 for WM_ALL_ and 4.14 for Vb_ALL_, so clusters and hiatuses identified in this study are very specific to Vb cyclones and do not apply to WM cyclones to the same extent. The ratio is highest for Vb‐GoG in terms of the subtypes. The ratio (ii) is 1.13 for WM_ALL_ and 1.65 for Vb_ALL_, so the synchronization with a negative or positive phase of the NAAO is again much stronger for Vb than for WM cyclones in general. Nissen et al. (2010) found a higher ratio of about 1.20 for WM‐cyclones (compared to 1.13 above), however restricting their analysis to the extended winter season from October to March and to cyclones identified at SLP. As a third test, the ratio of the mean annual number of cyclones during hiatus H7 as compared to the remaining years is 0.85 for WM_ALL_ and 0.63 for Vb_ALL_, hence the big hiatus (1988–1996) is not only a strong feature of Vb_ALL_, but is also apparent for WM cyclones in general. These findings are robust against the choice of threshold of cyclone intensity, although the relationship between the NAAO and the number of cyclones gets slightly stronger with increasing cyclone intensity threshold.

#### 3.4.2 Short‐Term Response in the Event of Vb

**Figure 8:**
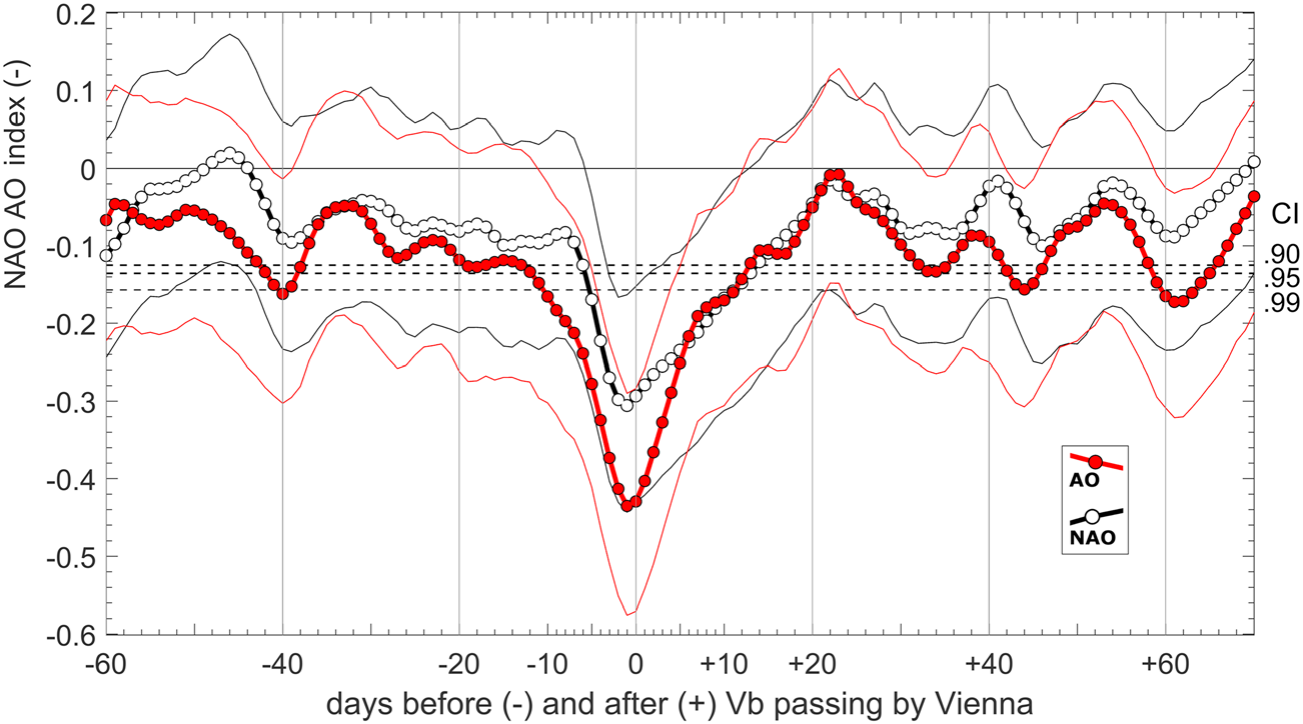
Evolution of the NAO and AO indices over 4 months as an average over 557 Vb events. The central point is defined as the day d_0_ when the cyclones reach the crossing line (CL in Figure 1). The dashed lines show the 0.90, 0.95, and 0.99 percentile CI, calculated from 10^6^ Monte Carlo experiments. For each experiment 557 time‐intervals (length = 130 days) from the period 1959–2015 are chosen randomly, the mean evolution of the NAO/AO is calculated and the lowest value out of the 130‐day period is determined. The thin solid lines indicate 0.90 and 0.10 percentiles from 10^6^ ordinary bootstrap experiments using 80 out of the 557 Vb events. CI = confidence interval.

To better understand the large‐scale processes during Vb events, the mean temporal evolution of the NAO and AO indices is shown in Figure 8 as an average over all 557 Vb events. The timeline starts from 60 days before and ends at 70 days after d_0_, the point in time when the cyclone is closest to Vienna (48°N/16°E, see Figure 1). This location was chosen to also include cyclones that emerge just before crossing CL at 47°N.

**Figure 9:**
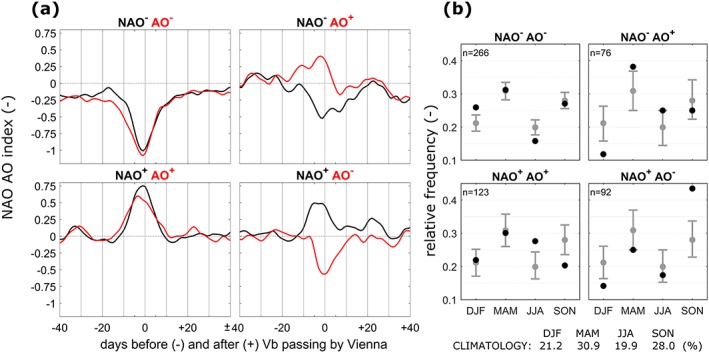
(a) Mean evolution of the NAO and AO indices for 3 months similar to Figure 8 but for the four NAO/AO modes of Table 5. (b) Seasonal relative frequency of Vb cyclones (black dots) compared to their climatology without stratification (gray dots and numbers at bottom). Gray whiskers indicate tenth and ninetieth percentile confidence intervals calculated from 10^6^ ordinary bootstrapping resamples of size = n without replacement by randomly shuffling the underlying dates. The number of events for each group is given in the top left corners. NAO = Northern Atlantic Oscillation; AO = Arctic Oscillation; DJF = December‐January‐February; MAM = March‐April‐May; JJA = June‐July‐August; SON = September‐October‐November.

From 40 to 10 days before the cyclone crosses the CL line, the AO and the NAO are generally in a negative phase, although not significantly low. A few days before Vienna is reached, AO and NAO drop dramatically, even when averaging over the 557 events. The drops last for about 23 and 19 days, respectively. The drop is more pronounced for the AO and starts as early as 10 days before the minimum. Obviously, a strong imprint on the LAC appears during Vb events, realized as a positive pressure anomaly at different atmospheric levels (Z500 and Z1000) at higher latitudes, even far upstream of the WM and Central Europe over the North Atlantic Ocean. After the minimum, the AO and NAO rise steadily, but remain at a low level for 2 weeks and reach a similar level to the one observed before the drop after about 17 days. This time span corresponds very well with the average interarrival time of *T* = 17.0 days of the 11 cyclone cluster periods found above. This similarity underpins the strong relation between the atmosphere over the Northern Atlantic and over Europe in the event of Vb. Although the mean drop of the NAAO shown in Figure 8 is significant, it is just half the standard deviation and therefore not very strong. When analyzing individual Vb events, it turns out that the NAAO has occasional strong positive peaks. These may explain why the mean NAAO is not negative for C8 as expected (Figure 7). To shed more light on this issue, the temporal evolution of the AO and NAO is further stratified into four groups, as a function of the sign of the NAO and AO at the time d_0_ (5‐day centered average). The largest number of cyclones (266 events or 47.8%) occurs when both NAO and AO are negative (Vb‐mode 1). If only mode 1 events are considered, the drop of both the NAO and AO is considerably stronger than for all events and reaches one times the standard deviation (Figure 9a, top left).

Above it has been speculated that the high number of Vb winter cyclones before 1971 may be connected to a dominant negative phase of the NAAO observed during the 1960s which is in line with, for example, Delworth et al. (2016). This notion is further supported by the seasonal number of Vb cyclones for each of the four NAAO Vb‐modes as compared to its climatology without stratification (Figure 9b). Vb cyclones inducing NAO^−^AO^−^ conditions (mode 1) show a significantly higher number of cyclones during winter (DJF) and a lower number during summer (JJA) so mode 1 appears to be a feature of the winter half year. As a consequence, the hypothesized positive feedback between the LAC over Europe and a high frequency of Vb events should also be interpreted as a main feature of the winter. This is in agreement with an equatorward shifted eddy‐driven jet stream and storm tracks over the Northern Atlantic observed under NAO^−^ in winter (Athanasiadis et al., 2010; Wettstein & Wallace, 2010; Woollings et al., 2010).

**Figure 10:**
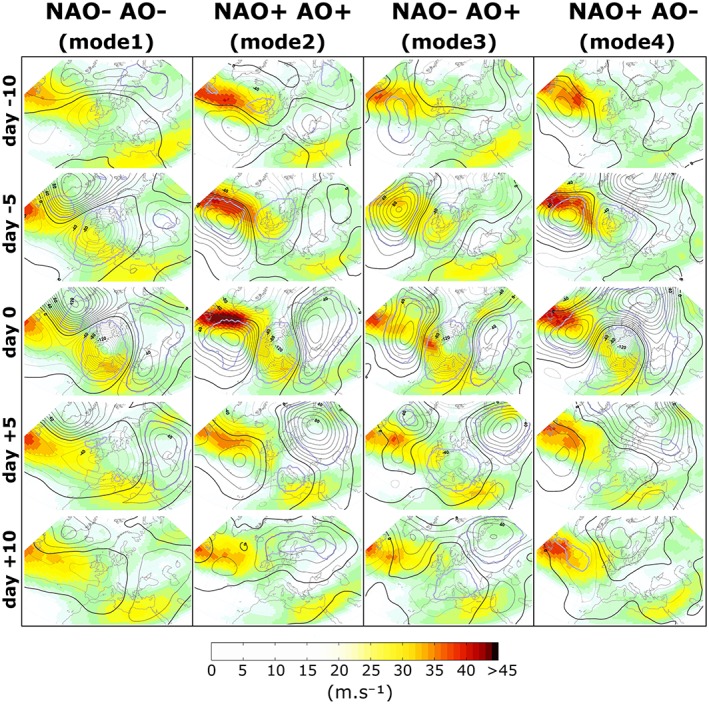
Temporal evolution of the large‐scale atmospheric circulation during Vb events from 10 days before to 10 days after crossing line Crossing Line at 47°N. Anomalies of 500 hPa geopotential height are shown as black contours (gpm), upper level (300 hPa) wind speeds are shown as color shading. Significance (p = 0.01 and p = 0.99 resp.) is indicated through gray solid contour lines for all anomalies exceeding ±20 gpm, calculated from 10^4^ Monte Carlo experiments. At day 0, a coupling of the eddy‐driven polar jet stream and the subtropical jet stream can be seen over the Western Mediterranean. In all modes, a strong impact of the Vb events on the large‐scale atmospheric circulation can be seen, with large positive pressure anomalies developing downstream around Scandinavia (modes 2–4).

For the 123 cyclones of the Vb‐mode 2 (NAO^+^ AO^+^) conditions, both NAO and AO exhibit a clear positive peak at d_0_ (Figure 9a, bottom left). These cyclones are more frequent during the summer (JJA) as compared to climatology. In absolute numbers, Vb‐mode 2 is more clearly a phenomenon of spring/summer and less so of autumn/winter. This mode could therefore drive the prime April/May frequency peak of Vb events, apart from the secondary peak found in late autumn (Hofstätter & Chimani, 2012; H18). The temporal evolution of mode 2 is very different that of mode 1, with a much stronger positive pressure anomaly developing over Scandinavia/W‐Russia (d_+5_ in Figure 10). In combination with the very pronounced high pressure anomaly over the Azores Islands the jet stream splits over the United Kingdom and is weakening faster over the WM as compared to mode 1.

The other two modes (Figure 9a, right) account for 13.6% (NAO^−^ AO^+^) and 16.5% (NAO^+^ AO^−^) of all Vb events (Table 5). These types show a peak of the NAO and a drop of the AO at the same time, which are weaker than modes 1 and 2. Most importantly, 40 out of the 92 Vb events associated with mode 4 (NAO^+^ AO^−^) occur in autumn, the season when the mean meridional temperature gradient, and hence circumpolar westerlies are increasing again, the polar‐front jet is shifting back southward and when the Mediterranean sea surface temperatures are still high.

**Table 5:** Partitioning of the 557 Vb Events into Four Modes by the Sign of the NAO and AO Indices at the Time the Cyclones Crossed the Confidence Interval Line

Vb mode	NAO index	AO index	Number of events (n)	Relative number (%)
1	−	−	266	47.8
2	+	+	123	22.1
3	−	+	76	13.6
4	+	−	92	16.5

*Note*. NAO = Northern Atlantic Oscillation; AO = Arctic Oscillation.

As a further interesting result, the average date for mode 1 and mode 4 Vb events differs significantly from the overall mean (16 April 1986) as shown in Table S2, with mode 1 events occurring earlier in time (the mean is 10 December 1984), in contrast to mode 4 events occurring later in time (the mean is 10 March 1988) than expected by random cyclone occurrence. The 60% of the winter (DJF)‐mode 1 events are found during the first 20 of the 57 years. The winter (DJF)‐mode 1 events might be related to the predominantly negative phase of the winter NAO observed during the years 1955–1970 (Iles & Hegerl, 2017), as this mode is associated with the cold season (Figure 9b). Mode 4 events are not only most frequent in Autumn (about 42%) but also 53% of the (September‐October‐November)‐mode 4 events occurred during the last 17 of the 57 years. This may be due to the strong increase of WM sea surface temperatures of about +1 °C after 1990 (Ionita et al., 2017; their Figure 10d), apart from possible decadal background variations of the LAC, leading to a higher frequency of Vb‐mode 4 cyclones during autumn in recent years.

Finally, we present the temporal evolution of the 500 hPa geopotential height anomaly, averaged over 263, 127, 75, and 92 days for the respective modes 1–4, as a sequence from 10 days before to 10 days after the cyclones crossing the CL line (Figure 10). For this analysis, the time series of GPH500 are first detrended and the annual cycle is removed at each grid point (Barriopedro et al., 2010). In addition, wind velocity is shown at the level of 300 hPa in color shading, indicating the mean strength and position of the jet stream. The partitioning of the Vb events into four groups is based on the sign of the NAO and AO at time d_0_ (Table 5).

For mode 1 slightly negative NAO conditions are present 10 days before d_0_ (d‐10), with a negative pressure anomaly found as precursor of the subsequent Vb event located over Western Europe. This anomaly subsequently develops into a major baroclinic trough, leading to a strengthening and southward shift of the eddy‐driven jet stream into the WM at d‐3. Next, a superposition of the eddy‐driven polar jet stream and the subtropical jet stream can be seen over the WM Sea at d_0_. In fact, this superposition occurs in all of the four modes and already starts (not shown) at d‐4 over the Balearic Islands (mode 1), at d‐3 over the Gulf of Genoa (mode 2), at d‐2 over Sardinia (mode 3), and at d‐1 over the Gulf of Lyon (mode 4). However, for mode 1 the coupling appears to be strongest, continues even until d + 10 (d + 5 for modes 2–4) and regains the same strength as at d‐10. The superposition imposes a zone of increased baroclinity and wind speed, favoring the development of cyclones through an enhanced ageostrophic circulation (Christenson et al., 2017) for another 10 days or more in the WM for mode 1.

Apart from the superposition of jets, a pronounced high pressure anomaly develops over Scandinavia in modes 2–4, inducing a blocked flow situation over Europe and a split and/or tilt of the polar jet stream. The positive anomaly remains until d + 10 in all these modes, so lasts for at least 1 week after the Vb event. A strong link between European/Scandinavian blocking and a discontinued or branching jet stream over the North Atlantic has been found by Madonna et al. (2017) and classified as the fourth main North Atlantic eddy jet regime (mixed type M_4_, see their Figure 6g) beside the mean zonal framework (Woollings et al., 2010) in accordance with the flow situation shown in Figure 10. This figure also shows that in the event of Vb a high pressure anomaly over Scandinavia either is rapidly developing (modes 2 and 3) or is clearly strengthened (mode 1) between d0 and d + 5. Apart from wave breaking and isentropic advection of air comprising low potential vorticity, adiabatic heating through latent heat release from ascending air has been identified as another important mechanism for blocking occurrence (Pfahl et al., 2015). The poleward advection of warm and moist air through the warm conveyor belt (Madonna et al., 2014) from Southern/Eastern Europe during Vb events (Grams et al., 2014) could therefore be a crucial ingredient fueling Scandinavian blocking apart from anticyclonic wave breaking (Masato et al., 2012).

## 4 Conclusions and Outlook

This paper explores the temporal characteristics of Vb cyclone occurrence based on cyclone tracks identified at the atmospheric levels of Z700 and SLP, using JRA‐55 reanalysis data for the period 1959–2015. Vb cyclones typically emerge in the WM and propagate to the Northeast into Central Europe. The cyclones are classified into four Vb‐subtypes, denoted as GoG, DiN, IbA, and OuT (Table 1), based on the region they are first detected. The temporal characteristics are assessed by analyzing changes of the cyclone occurrence rate as well as by identifying individual cluster and hiatus periods as compared to a random Poisson point process. The relationship between the LAC and Vb occurrence rate is investigated by examining selected teleconnection indices, in particular the NAO index at Z500 and the AO index at Z1000. Finally, the temporal evolution of the geopotential height and upper level wind speeds over Europe and the Atlantic is analyzed to identify linkages between Vb cyclones and the LAC. From the analyses, five major findings have been obtained.

1. The risk of Vb occurrence has decreased significantly between 1959 and 2015 from 12.4 to 9.4 events per year, which is mainly due to the very high number of Vb cyclones developing over the Iberian Peninsula or North Africa during the winters before 1971. Between 1971 and 2015 the risk has remained rather constant for all Vb subtypes, apart from decadal variations, which is in line with trends of other WM cyclones (Lionello et al., 2016; Maheras et al., 2001; Nissen et al., 2014). The current rate of subtype Vb‐GoG cyclones developing on the southern Alpine lee side is close to its long‐term average. This is a counterintuitive result as midlatitude storm tracks are expected to shift poleward due to climate change (Seneviratne et al., 2012; Woollings & Blackburn, 2012) ahead with a weakening of the meridional temperature gradient and baroclinity over the Northern Atlantic (Wang et al., 2017) and near surface air temperatures have already increased by 0.9 °C since 1950 over the Northern Hemisphere (Jones et al., 2012; Morice et al., 2012; Osborn & Jones, 2014). This suggests that either internal variability is considerably large compared to externally driven changes in recent decades, or other processes are compensating for climate change effects. Vb cyclogenesis is strongly linked to the upper level atmospheric dynamics interacting with major orography in a low‐level baroclinic environment in the WM (e.g., Maheras et al., 2002; Trigo et al., 2002). The increase of WM sea surface temperatures of about +1 °C after 1990 (Ionita et al., 2017; their Figure 10d) appears as a plausible explanation for a sustained Vb cyclone occurrence rate.
2. Vb cyclones do not occur fully randomly according to a Poisson point process, that is, they arrive either clustered or more regularly at times. Clustering is very prominent in the case of Vb‐GoG cyclones and is particularly strong in summer for this type. On the other hand, clustering also occurs in winter, but primarily for Vb‐IbA cyclones that develop over the Iberian Peninsula or the North African Coast. This major difference could be related to differences in the formation processes of clusters in different seasons between the Iberian Peninsula and the Genoa region, but further research is needed on this matter. Eleven well‐separated and distinct clusters as well as 11 hiatus periods are identified, with average occurrence rates of 21.5 and 5.2year^−1^, respectively. As Vb cyclones are strongly related to large‐scale heavy precipitation over Central Europe (H18; Messmer et al., 2015; Nissen et al., 2013) these phases are expected to correspond to flood‐rich and flood‐poor periods in catchments mainly affected by Vb cyclones (Hall et al., 2014). The duration of the Vb clustering periods are typically between 0.5 and 1.5 years (Table 4), in consistency with clustering found in German flood time series on an intra‐annual to interannual time scale (Merz et al., 2016).
3. Superposition of the eddy‐driven polar jet stream and the subtropical jet stream over the WM is identified as a prominent feature at the onset of Vb events (Figure 10). Superposition events (e.g., Christenson et al., 2017) are usually marked by a vanishing latitudinal separation and vertical merging of the jet axes, leading to increased horizontal wind speeds (e.g., Reiter & Whitney, 1969). At the same time, stratospheric and tropospheric baroclinity is combined within a narrow zone, resulting in high available potential energy and a further intensified ageostrophic circulation steering cyclogenesis (Handlos & Martin, 2016). Apart from major synoptic eddies developing over Western Europe and amplifying in the WM, the superposition of the jet streams over the WM seems to invigorate Vb cyclogenesis. Superposition is more likely if the latitudinal separation of the jets is small, which tends to occur during negative NAO phases. Superposition of the jet streams was also found to occur over the WM/East Atlantic at 40°N during a negative phase of the NAO (Woollings et al., 2010) and to be associated with Greenland blocking events (Woollings et al., 2008).
4. Large‐scale atmospheric patterns, specifically the NAO (at Z500) and AO (at Z1000), appear to be synchronized with the occurrence of Vb cyclones. During months of Vb cyclone occurrence, NAO and AO are much more negative than in months without Vb cyclones. The Scandinavian pattern however is more positive (Figure S2) in this regard. Ten of the 11 clusters of Vb events occur during a negative phase of the NAAO (Figure 6) which is significantly more negative than random occurrence (Figure 7). On the one hand the latitudinal position of the polar jet stream appears to be a responsible mechanism explaining this relationship, as its location is shifted to more southern latitudes under negative NAO conditions (Woollings et al., 2010), in correspondence with high‐latitude blocking at the same time (Woollings et al., 2008), thus pushing cyclogenesis toward the WM (Nissen et al., 2010, 2014). We find this mechanism to be most relevant for Vb occurrence during the winter half year (mode 1 events). Cyclone clusters over NW‐Europe, in contrast, are associated with a persistent and intensified eddy‐driven jet stream located at 50°N over the NE‐Atlantic (Pinto et al., 2014, 2016) in coherence with a positive phase of the NAO (Mailier et al., 2006). On the other hand, the relation between the mean state of the NAAO and Vb frequency (chapter 3.4.1) is much stronger for those systems following the Vb‐track (1.65), as for cyclones developing in the WM in general (1.13). Obviously, the upper level atmospheric flow over Central Europe is changed into a state favoring the propagation of a series of cyclones from the WM to the northeast on track Vb. Indeed, negative NAO conditions are associated with a weak zonal flow (Woollings et al., 2010) and dominant meridional flow patterns over Europe (Dünkeloh & Jacobeit, 2003) supporting this finding. However, this does not entirely explain the even higher ratio of Vb cyclones between clusters and hiatuses with 4.14 for Vb‐ALL. The very high arrival rate during clustering periods could be caused by a combination of (i) a sustained large‐scale atmospheric state which forces cyclones from the WM to move along track Vb and (ii) by a self‐exciting process in which the Vb event itself reinforces the LAC into a state that favors the development (genesis) of a successive Vb cyclone (positive feedback) on top of background forcing from the atmosphere over the Northern Atlantic Ocean. Further research is needed on this issue.

Finally, some specific limitations of the study are mentioned. The NAAO/Vb‐rate relationship applies on average, but not every single cluster or hiatus period follows this relationship. This may be due to (i) an opposed state of the NAO or AO occurring from time to time, leading to blurring in the combined and temporarily averaged NAAO index, (ii) in some instances the Vb cyclones are weak and limited in extent, so have no strong impact on the LAC, and (iii) Vb events are rare so this limited number of days cannot fully account for all the NAO or AO variability. Also, cluster or hiatus periods were not observed in a number of instances although the state of the NAAO suggested their occurrence. Local factors may also be important for cyclogenesis in the WM apart from the large‐scale forcing (e.g., Aebischer & Schär, 1998; Trigo et al., 2002), and 22% of the Vb events involve a positive peak of the NAO and NAO (mode 2 events) so Vb occurrence is not strictly tied to a negative phase of the NAAO, especially in the summer. It should also be noted that the identification of Vb cyclones is restricted to all systems moving northward into Central Europe between 12 and 22°E at 47°N, so some cyclones might have been missed if they passed outside the line or had already dissipated in the WM.

Atmospheric reanalysis data are potentially affected by homogeneity issues or temporal inconsistency, especially in data sparse regions after 1979 (e.g., Wang et al., 2016). Concerning JRA‐55, observational data as synop, ship, buoy, and radiosondes as well as tropical cyclone wind retrievals are used back to 1958. In 1973 a large number of aircraft data, as well as pseudo‐observations of surface pressure and reprocessed satellite data, were incorporated. Other satellite data were assimilated from 1979 onward, complemented by more satellite data between 1995 and 2007. The quality of JRA‐55 therefore continuously improves over time, as indicated by the RMSE of Z500 (48‐hr forecast score) averaged over the Northern Hemisphere continuously decreasing from 25 to 15 gpm between 1962 and 2012 (Kobayashi et al., 2015). The integration of additional data (quantity and types) over time, however, did not result in a statistically significant (α = 0.05) change point for the consecutive seasonal cyclone count averaged over the Northern Hemisphere before 1997 in JRA‐55 (Wang et al., 2016; Tables 2 and 3 therein). Only for the variance of mean cyclone intensity two breakpoints were found at the end of 1972 (a minor one) and 1997 (a major one) in case of deep systems. There is therefore reasonable confidence that the high number of Vb cyclones found between 1959 and 1971 do not stem from data inhomogeneity, especially as the integration of satellite data after 1973, 1979, and 1995 would not result in a decreasing number of detected cyclones over time as more information was added.

Finally, the number of cyclones is usually higher with increasing horizontal resolution between different reanalysis data (e.g., Neu et al., 2013; Tilinina et al., 2013; Wang et al., 2016), mostly due to the recognition of weak and smaller cyclones (Raible et al., 2008; Rohrer et al., 2018). The limited spatial resolution of JRA‐55 is therefore expected to hamper (i) the recognition of developing cyclones in their early stage, potentially leading to small number of ambiguous cases when classifying Vb‐tracks into the four subtypes (Figure 1 and Table 1), and (ii) the recognition of small/weak systems in general, especially in the vicinity of the Alps or Pyrenees. However, temporal trends and interannual variability is usually much less affected by resolution issues (e.g., Lionello et al., 2016; Wang et al., 2016) and should therefore not be an issue in this study, also because atmospheric data were filtered by a spatial low‐pass filter.

## Supporting information

Supporting Information S1
