# Supplementary material for: Vb Cyclones Synchronized With the Arctic‐/North Atlantic Oscillation
Source: J Geophys Res Atmos. 2019 Mar 26;124(6):3259–78. doi: 10.1029/2018JD029420 (PMC6559292; doi:10.1029/2018JD029420)
Supplement: Supplementary file 1 — Supporting Information S1 [file JGRD-124-3259-s001.docx]

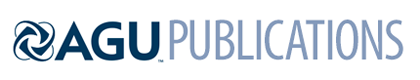


*[Journal of Geophysical Research: Atmospheres]*

Supporting Information for

Vb cyclones synchronized with the Arctic-/North Atlantic Oscillation

M. Hofstätter^1^ and G. Blöschl^2^

^1^Department of Climate Research, Central Institute for Meteorology and Geodynamics, Hohe Warte 38, 1190 Vienna, Austria. ^†^ Vienna Doctoral Programme on Water Resource Systems, Vienna University of Technology, Austria

^2^Institute of Hydraulic Engineering and Water Resources Management, Vienna University of Technology, Austria.

**Contents of this file**

Figures S1 to S2

Tables S1 to S2

**Introduction**

Supplementary information includes two figures S1 and S2 as well as two tables S1 and S2.

Figure S1 shows a comparison of the sample and theoretical cumulative distribution function for the Vb interarrival times. For a Poisson point process, interarrival times T should be exponentially distributed which is examined in Figure S1. Additionally, the Anderson-Darling test is used to test whether the sample comes from an exponential distribution (H0: “interrarrival times are iid exponentials”). The test statistics (*AnDa*) at the 5% significance level are given at the upper left corners of the panels. The associated p-value is the probability of observing *AnDa≥cv* under H_0_

In figure S2, the mean monthly state of selected teleconnection indices ($\bar{TCI}_{k}$) is shown, stratified by the monthly count *k* of Vb cyclones into five groups (*k*=1,2,3,4, and 5 or more). The mean of the respective TCI for all months without Vb cyclones ($\bar{TCI}_{0}$ at the top of Figure 7) is used as a reference and subtracted from $\bar{TCI}_{k}$. The straight black lines, estimated by linear least absolute deviations regression, indicates that certain TCI’s are more strongly negative (positive) with an increasing (decreasing) number of Vb events per month.

Table S1 shows a parametric trend test (Cox-Lewis U-test) to test for monotonic trends in the occurrence of Vb cyclones in the period 1959-2015. It also includes the lag-1 autocorrelation coefficient of the interarrival times.

Table S2 shows the mean date for the four modes of Vb occurrence in comparison to the overall mean date (1986-04-16) including percentile-confidence intervals.


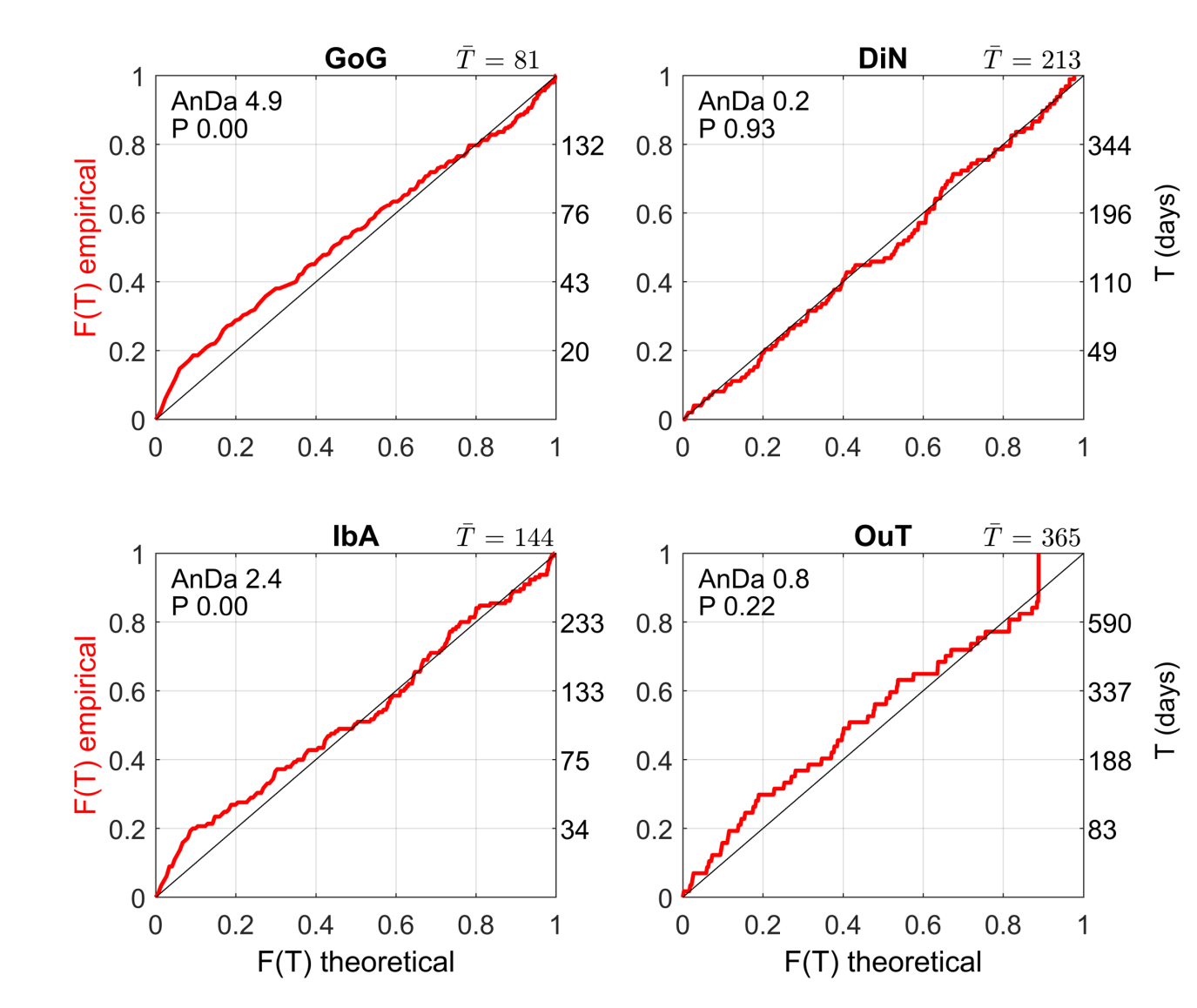


Figure S1. Cumulative distribution functions (cdfs) of observed interarrival times T (red) plotted against exponential cdfs (black). The Anderson Darling (AnDa) test statistics and the associated p-values (α=0.05) are given at the upper left corners of the panels. as well as the mean interarrival times $\overline{\mathbf{T}}\mathbf{=}\boldsymbol{\lambda}^{\mathbf{-1}}$ (days) on top. The associated p-value is the probability of observing AnDa≥cv under H0 with the critical value cv=1.31.


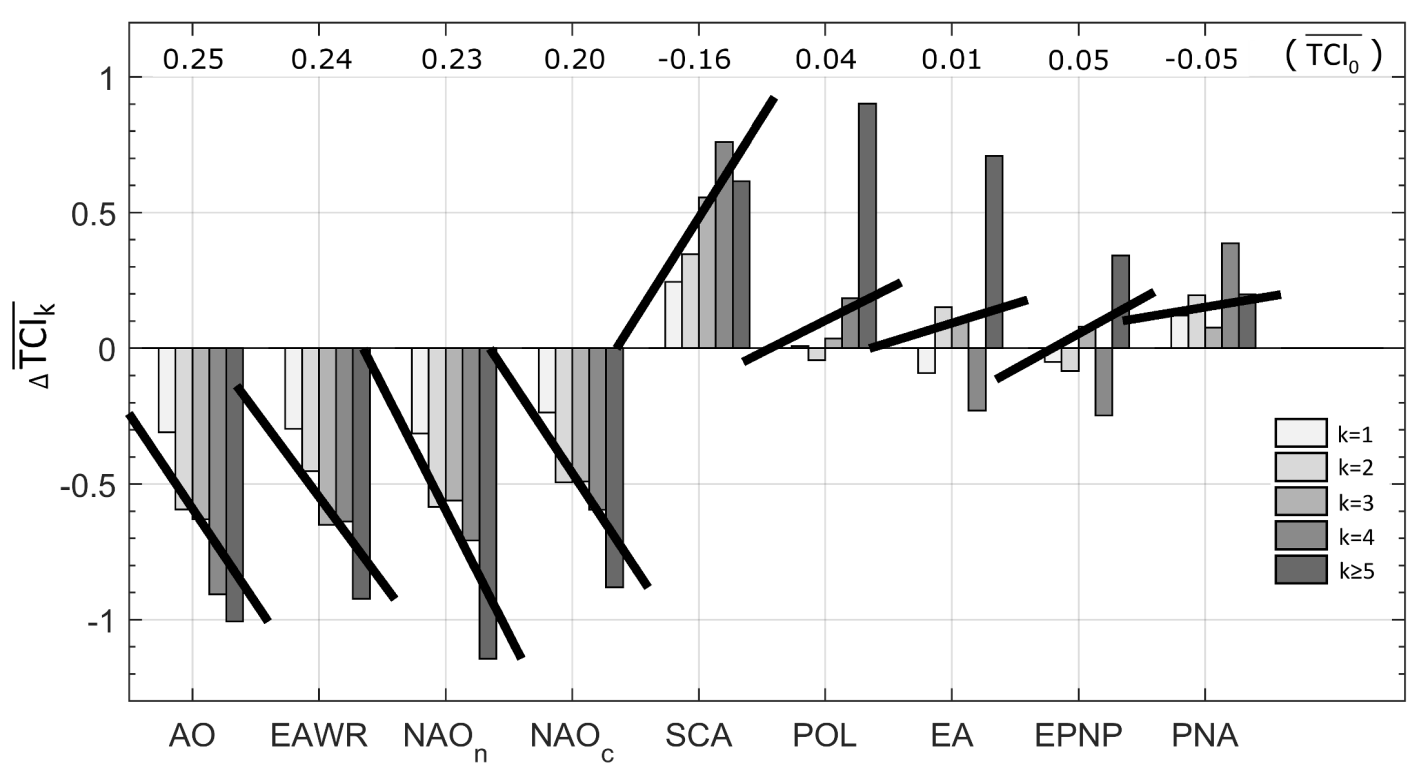


**Figure S2**. Mean monthly teleconnection index $\Delta\bar{TCI}_{k}$ as an average over those months with *k*=1,2,…,5 or more Vb events per month: $\Delta\bar{TCI}_{k}=\bar{TCI}_{k}-\bar{TCI}_{0}$ with the reference state $\bar{TCI}_{0}$ for months without Vb events (*k*=0) for the AO, EAWR, NAO_n, NAO_c, SCA, POL, EA, EPNP and PNA patterns. Numbers on top indicate reference state. Classes *k*= 1, 2, .., 5 consist of 230, 78, 33, 10, 4 months, class *k*=0 of 329 months. The straight black lines, estimated by linear least absolute deviations regression, indicates dependence between the number of Vb events per month and the strength of the respective index.The AO, NAO and EAWR patterns are in a negative state during months when Vb cyclones occurred ($\bar{TCI}_{k}<0$) but are in a positive state otherwise ($\bar{TCI}_{0}>0$).

Table S1 (a-c). Cox-Lewis U-statistic to test for monotonic trends in the occurrence of Vb cyclones in the period 1959-2015, using a parametric regression model (Cox and Lewis, 1966, p. 47) with H_0_ = “no trend; λ=constant”. (c): Lag-1 day correlation for the square root-transformed and linearly detrended interarrival times. IbA_wi refers to winter cyclones from the Iberian peninsula and the North African Coast, for the other abbreviations see Table 1. Bold print indicates p<0.05.

|  | Vb-cyclones | ALL | ALL except IbA_wi | GoG | DiN | IbA | OuT |
| --- | --- | --- | --- | --- | --- | --- | --- |
| (a) | U-test statistics | -1.67 | -0.87 | 0.10 | -1.67 | -2.02 | -0.01 |
| (b) | p-value (one-sided) | **0.05** | 0.19 | 0.46 | **0.05** | **0.02** | 0.50 |
| (c) | R_1_: lag-1 correlation of T | 0.05 | / | 0.02 | 0.13 | -0.09 | -0.18 |

**Table S2.** Mean date for mode 1-4 Vb events and confidence intervals from drawing 5x10^7^ random samples of size=n (shown in Table 5) with replacement for each mode from the given 557 Vb dates (at time d_0_). Mean dates outside the 0.10 or 0.90 percentile values are printed in bold.

| Vb mode | mean date | 0.10 percentile | 0.50 percentile | 0.90 percentile |
| --- | --- | --- | --- | --- |
| 1 | **1984-10-12** | 1984-12-13 | 1986-04-15 | 1987-08-17 |
| 2 | 1988-05-23 | 1984-04-27 | 1986-04-15 | 1988-04-04 |
| 3 | 1985-07-24 | 1983-10-15 | 1986-04-15 | 1988-10-18 |
| 4 | **1988-10-03** | 1984-01-05 | 1986-04-15 | 1988-07-26 |
